# Sociodemographic Correlates of Parental Co-Participation in Digital Media Use and Physical Play of Preschool-Age Children

**DOI:** 10.3390/ijerph18115903

**Published:** 2021-05-31

**Authors:** Elina Hasanen, Henriikka Koivukoski, Lauri Kortelainen, Hanna Vehmas, Arja Sääkslahti

**Affiliations:** Faculty of Sport and Health Sciences, University of Jyväskylä, 40014 Jyväskylä, Finland; henriikka.m.koivukoski@jyu.fi (H.K.); kortelainen.lauri.j@gmail.com (L.K.); hanna.m.vehmas@jyu.fi (H.V.); arja.saakslahti@jyu.fi (A.S.)

**Keywords:** young children, physical activity, digital media use, parental co-participation, sociodemographics, guidelines

## Abstract

Young children’s digital media use and physical activity have gained attention in recent research. Parental co-participation has a major impact on children’s health consequences. This study addressed a gap in the research by investigating daily parental co-participation in children’s digital media use and physical play, using the family ecological model theoretical framework. The participants in this nationally representative cross-sectional study were 2512 Finnish parents with two- to six-year-old children. Parents completed a questionnaire. Sociodemographic correlates of co-participation and of the awareness of guidelines regarding co-participation and correlation between co-participation in digital media use and physical play were analysed. Parental co-participation in physical play and digital media use correlated positively. Lower parental age, male parental gender, Finnish and Swedish languages, a fewer number of children, and a male child gender were associated with more co-participation in one or both activities, and parental female gender and low family income were associated with more awareness. The awareness of guidelines was not associated with co-participation in digital media use. There were sociodemographic differences in parental co-participation. From a health counselling perspective, parents may benefit from national recommendations on digital media use and physical activity, but adherence to guidelines depends on the family context.

## 1. Introduction

Early childhood is a foundational period of life for the development of health behaviours [1,2]. Regular physical activity (PA) has favourable health effects in young children [3,4], and PA habits established in early childhood track in later childhood [5,6] and, subsequently, in adolescence and adulthood [7]. Sedentary behaviour habits also tend to track from childhood to later life [6,8,9]. Worldwide, children’s PA does not reach the recommended daily level [10], and obesity deriving from sedentary behaviours of the western lifestyle has been considered a major threat to health during childhood [11,12,13]. Screen time includes various types of digital media use (DMU) in various contexts, but excessive sedentary screen time has been associated with several risk factors for poor health in childhood [14,15,16], such as low PA level and increased BMI [17]. These risk factors have long-term significance and are related to, for example, problems in children’s social skills [18] and socio-emotional development later on [19]. The rapid changes in digitalisation, such as increasing DMU [20], require up-to-date research on children’s behaviour and the factors influencing them [21].

The family setting has a major influence on preschool-age children’s daily PA and DMU [22,23]. Studies have shown that parents play a key role in supporting the development of healthy behaviour patterns [24,25,26] and have an especially strong role during early childhood [27]. One of the influential means of support is parental co-participation [28], that is, parents engaging in the activity with their child [29]. Parental co-participation has been associated with health-enhancing effects with regard to preschool-age children’s PA and DMU. Co-participation in PA has been associated with the likelihood of the child engaging in regular PA [30,31,32] and a positive effect on parents’ PA [33,34], as well as outcomes such as improved family relationships [35]. In young children’s DMU, the health-enhancing effects of parental co-participation are often recognised as mitigants of negative cognitive and social impacts of DMU [21,36,37], such as poor psychosocial development [19] or impaired language development [38], but also as promoters of new learning opportunities [39]. Moreover, social inequalities in children’s DMU have been linked to differences in parental mediation [40]. In order to have the aforementioned positive health impacts on children’s health and development, parental co-participation should ideally be active in nature, meaning, for example, the inclusion of discussion and teaching while engaging in DMU. This type of active mediation has been distinguished from the mere restriction of DMU [41,42] or passive co-use [43]. Today, parental co-participation in young children’s daily activities is mostly sedentary by nature [44], such as the co-use of digital and non-digital media [41]. Parental co-participation in young children’s PA seems to be mostly light activity and little in daily amount [44,45], as well as irregular [34] but, nevertheless, highly valued by the parents [46]. The evidence for the correlation between parental co-participation in children’s PA and a higher amount of children’s PA [24,30,31,47] suggests the importance of investigating daily parental time use. While engaging in children’s activities, parents transmit their attitudes and values, and, thus, health-related behaviours, to their children [23,24,25]. There is a lack of knowledge on daily parental co-participation in PA and DMU in two- to six-year-old children. Concerning DMU, it is necessary to focus on active parental co-participation due to its favourable effects on health and development.

The present study investigated parental co-participation in the family setting using the family ecological model (FEM) [48] theoretical framework. The FEM emphasizes the role of contextual factors in shaping parental behaviours, which then influence child and family health outcomes. Likewise, the family is emphasized as an intervention target instead of an individual. Among the contextual factors, family ecological factors include sociodemographic factors such as family structure, child-specific characteristics and community factors. Previous research has found statistical differences in parental co-participation in DMU based on parental gender [41,49], age [41] and education [31,41]; child age [41,49] and gender [41]; and family cultural background [41], income and structure [50]. Research has also found country-specific cultural differences [51]. Parental co-participation in PA varies according to child and parental gender [52], child age [53], parental age [49], family structure [49] and family income [53]. Due to differences in study designs, included factors and, consequently, dissimilarities between findings, there lacks a consistent picture of the role of the relevant sociodemographic factors that shape parents’ daily co-participation in young children’s activities. Moreover, according to the FEM, family ecology shapes parental behaviour by influencing the family social and emotional context, including knowledge and beliefs about healthy behaviour [48]. Guidelines, such as those of the American Academy of Pediatrics (AAP), which recommend co-participation in children’s DMU [54], influence knowledge and beliefs and, thus, parental co-participation. However, according to the FEM, contextual factors play a significant role in cognition. Research has found sociodemographic differences in the awareness of PA recommendations [55], as well as differences in co-participation based on parental competence and media skills [41,49,51]. Therefore, the factors influencing parental awareness of the guidelines recommending co-participation, as well as the association between awareness and co-participation, are worth investigating.

The aim of this study was to examine, first, how much time on weekdays and weekends children spend on DMU and on outdoor physical play and how much parents actively co-participate in these daily activities. The association between parental co-participation in DMU and physical play was also analysed. Second, this study investigated the correlations of sociodemographic factors with parental co-participation in the activities and with parental awareness of the guidelines regarding co-participation in DMU in early childhood. In addition, the association between parental awareness of the guidelines and parental co-participation in DMU was investigated.

## 2. Materials and Methods

### 2.1. Sampling and Survey Procedures

The study was a cross-sectional study of families with two- to six-year-old children living in Finland and was part of the research project International Ipreschooler Surveillance Study Among Asians and OtheRs (IISSAAR). The research data were collected in 2019 using a survey for parents. Preschool age in this study was defined as ages two to under seven years, the age when most children in Finland attend day care centres before beginning their obligatory school years.

Probability proportional to size (PPS) sampling was conducted, and stratums of municipalities were defined based on geographical location and spoken languages. Participants from the sample municipalities were recruited via municipal day care centres, which cater to the majority of Finnish children across social groups [56]. A total of 56 municipalities of the sample of 75 chose to participate, representing 18% of municipalities from all parts of the country. In addition, major national private day care chains were approached, and three major chains agreed to participate. All day care centres in the participating municipalities were contacted with a request for cooperation in disseminating the survey to parents. Out of the 1383 day care centres contacted, a total of 426 centres (30%) with approximately children chose to participate. As an exception, four municipalities and the private day care centres chose to disseminate the survey to all parents with children in all their day care centres. In order to access speakers of minority languages, the survey was offered in four commonly spoken languages: Finnish, Swedish, Russian and English. The original Surveillance of Digital Media Habits in Early Childhood Questionnaire (SMALLQ^®^) was translated from English to Finnish, Swedish and Russian, following the World Health Organization guidelines for cultural translation [57]. In order to better reach the Russian-speaking minority, information about the study was also disseminated via a nongovernmental organisation with contacts to this minority group. Additionally, a paper questionnaire was offered as an alternative to the online survey to promote equal chances of participation.

The survey took place from 11 November to 1 December, 2019. A reminder was sent to parents via day care centres a week to the response deadline. During data collection, weather conditions were normal for the season (late autumn/early winter, temperature varying from +8 to −17 °C, partly snowy).

### 2.2. Measurements

The research data were collected using the SMALLQ^®^, an online survey developed by Chia et al. [58] based on the conceptual understanding of digital media parenting by O’Connor et al. [59]. The questionnaire was targeted at parents, who were asked to report on only one child if they had more than one two- to six-year-old and included an option for another parent to respond on another child in the same age range in the family. The SMALLQ^®^ consisted of 25 questions, including questions on digital media habits of the child and the parent, nondigital media behaviour of the child and background information on the parent and child. In the SMALLQ^®^, DMU was defined as the time spent accessing content transmitted via television, computer, mobile devices, video game devices, blu-ray/DVD/CD/videotape players or intelligent/technological toys.

Child DMU was assessed separately for different purposes, including education/learning, entertainment, creating media, communicating and other activities, on weekdays and weekends (or other days off), using a seven-day recall. Respondents were advised to consider time outside of day care, for example, at home, and that total hours may be more than 24 h. The total child DMU was calculated by adding the time spent on the different digital media activities.

Children’s outdoor physical play was assessed alongside other nondigital media activities, such as indoor play and helping with simple household chores. Examples were “playing ‘hide-and-seek’ or ‘tag’, climbing, playing ballgames”. Similarly, a seven-day recall was used, and the measurement was performed considering time outside the day care centre.

Regarding parental co-participation in children’s DMU and physical play, parents were asked to estimate their percent engagement in their child’s total DMU time on weekdays and weekend days. An example was given: “interacting while watching videos together”. Co-participation in the child’s physical play was assessed similarly, including both indoor and outdoor physical play. Examples given included “playing ‘hide-and-seek’ or ‘tag’ together”.

In order to assess parental awareness of guidelines on DMU for children, in the SMALLQ^®^, parents were asked whether they were aware of the different guidelines on DMU for small children and if they followed those guidelines. The four variables included (1) limiting DMU for children younger than 2 years, (2) limiting screen time to 1 h per day for children 2–5 years, (3) introducing only high-quality educational programs for children 1.5–2 years and (4) co-watching or co-playing digital media with the child. In each variable, parents could choose one from the options “I am not aware”, “I am not aware BUT practicing”, “I am aware BUT do not practice” or “I am aware and practicing”. In this study, the fourth variable (co-watching with the child) was included and analysed, combining the response options in two categories, to those not aware and those that were aware.

A number of covariates were included in the study as sociodemographic factors. These were parental age; parental and child gender; parental education level and household income, indicating family socio-economic status; survey response language indicating parental cultural background; residential environment and family structure (number of adults, number of children aged 0–6 years and number of children aged 7–18). Parents who were native speakers of Finnish, Swedish or Russian chose to complete their survey in their native language. English was chosen as a survey language by respondents with 22 different native languages. Household income was assessed on a scale of seven, ranging from EUR 0–13,999 to 120,000 or more. Parental education level was in five categories: elementary/no formal education, secondary, vocational, bachelor’s degree and master’s degree. Residential environment was in accordance with the Finnish context of four categories and, for analysis, was combined to rural (countryside and town centres) and urban (cities and capital area).

### 2.3. Ethics

Ethical approval was obtained from the Nanyang Technological University (NTU). The protocol was consistent with the guidelines of the Finnish Advisory Board on Research Integrity [60]. At each step of data collection, a research permit was obtained, first, from early childhood education administrators of each municipality and day care chain and, second, from individually contacted day care centres. The study information letter for parents included the aims of the study, information on the voluntariness and anonymity of participation, a hyperlink to the privacy note and to the study webpage for more information on the research, information that there were no financial rewards for participation, a hyperlink and QR-code to the questionnaire, and information on the availability of the survey in a paper format. Parents could fill the survey after giving their informed consent. Data were collected online on a secure platform (Qualtrics) approved by the NTU.

### 2.4. Statistical Analyses

The averages of parental participation in DMU and physical play of a child were calculated as weighted averages from typical weekday and typical weekend day values. To describe the association between participation in physical play and DMU, the Pearson product-moment correlation coefficient was calculated with a 95% confidence interval.

As the percentage data were bounded and there were data values falling on boundaries (0% and 100%), linear regression or beta regression was not applicable. Instead, we used ordinal logistic regression analysis to examine the sociodemographic correlates of parental participation. For the analyses, the variables of parental participation were categorized as follows: values under 25%, over 25% but less than 75%, and greater than 75% were classified as “low”, “moderate” and “high”, respectively.

The association between the sociodemographic predictors and awareness of the guidelines on DMU for children was analysed using a binary logistic regression.

The covariates included in the models were language, age, gender and educational level of the parent/guardian, gross annual household income, number of adults in the household, number of zero- to six-year-olds in the household, number of 7–18-year-olds in the household, gender of the child and residential environment. Only the main effects of the covariates were used in the models. The assumption of proportional odds was met for both ordinal logistic regression models. In all models, the regression coefficients were converted to odd ratios (ORs) and are presented with their 95% confidence intervals. Additionally, *p*-values of the likelihood ratio tests for each covariate are shown. For statistical significance, *p* < 0.05 was used.

Analyses were conducted in R (R Core Team, 2020) using the package MASS [61].

## 3. Results

A total of 2512 parents agreed to participate in the study: 89% in Finnish, 7% in Swedish and 2% each in Russian and English (Table 1).

### 3.1. Characteristics of the Participants

Most of the parents’ responses were given by women (84%), and the children comprised an equal number of girls (48%) and boys (52%). The mean age of the parents was 36.2 years and the children, 4.7 years. Most of the parents had at least a bachelor’s degree (68%) and an annual household income of at least EUR 40,000 (77%). Most of the participants’ families were households with two adults (89%) and a minority of the participants were single parents (11%). Only 46 households included over two adults, and only three of the respondents were grandparents (not shown in table). It was most common to have one child aged 0–6 (61%) and no children aged 7–18 (63%). Table 1 shows the descriptive characteristics of the participants of this study.

### 3.2. Children’s DMU and Outdoor Physical Play

The mean DMU of children was 1.6 h (SD = 1.3) on a weekday and 2.2 h (SD = 1.4) on a weekend day, with no statistically significant difference between genders. The DMU increased with age.

Children spent 1.3 h (SD = 1.39) on a typical weekday and 1.8 h (SD = 1.06) on a typical weekend day on outdoor physical play. There was no statistical difference between age groups. However, boys spent slightly more time on outdoor physical play (weighted daily average 1.47, SD 1.2) than girls (1.31, SD 1.1, *p* = 0.002). Table 2 shows how much time children aged 2–6 spent in DMU and outdoor physical play on a typical weekday and weekend day.

### 3.3. Parental Co-Participation

Table 3 shows the proportions of time that the parents were engaged in their two- to six-year-old child’s DMU and physical play on weekdays and on weekends, categorized by child’s age.

On a typical weekday, parents were engaged in their child’s DMU for an average of 41% of the time the child used digital media. On a typical weekend day, the parental co-participation rate was, on average, 47%, slightly higher than on a typical weekday. Parental co-participation decreased with child’s age; for parents of two-year-old children, the co-participation rate was 57%, and for parents of six-year-old children, it was 31% on a weekday. Similarly, on weekends, younger children’s parents reported more participation in their child’s DMU compared to older children’s parents: the participation rate in DMU on a weekend day gradually decreased from 60% to 38% with an increase in child age.

On a typical weekday, parents were engaged in their child’s physically active play for 29% of the time on average; this proportion gradually decreased from 43% to 20% with an increase in the child’s age. On a typical weekend day, the average parental co-participation rate was 43%. Co-participation in physical play decreased as the child became older; it ranged from 57% with two-year-olds to 33% with six-year-olds.

There was a significant correlation between parental co-participation in DMU and physical play (r = 0.398 (CI 0.358–0.436)).

### 3.4. Sociodemographic Correlates of Parental Co-Participation

Table 4 shows associations between parental co-participation in the child’s DMU and sociodemographic covariates. There was a statistically significant difference between Russian and Finnish parents in co-participation in DMU (OR 0.314, CI 0.149–0.632). A higher age of a parent was associated with a lower co-participation in DMU (OR 0.973, CI 0.955–0.992). Additionally, if a child was a boy, the parent was more involved in DMU. Finally, a higher number of young or older children in a household was associated with less co-participation in DMU.

Table 5 shows associations between parental co-participation in physically active play and covariates. The Swedish-speaking parents were more likely to be involved in their child’s physical play compared to Finnish-speaking parents (OR 1.685, CI 1.137, 2.491). In addition, there was a negative association between a parent being female and participation in a child’s physical play compared to a parent being male (OR 0.620, CI 0.474, 0.812). Other statistically significant variables were parent’s age, child’s gender, number of young children and number of older children in the household, directions and OR magnitudes being similar to DMU analyses (see Table 4).

### 3.5. Parental Awareness of Guidelines

Parental awareness of the guidelines regarding co-participation in children’s DMU (“co-watching or co-playing digital media with the child”) was analysed in association with sociodemographic covariates. The regression coefficients of the logistic regressions analysis are shown in Table 6. According to the model, females were more aware of the guidelines than males were (OR 2.392, CI 1.780–3.215). People belonging to the middle income class (EUR 40,000–69,999) were significantly less aware of the guidelines than those of the lowest income class (OR 0.424, CI 0.194–0.850). There were no statistically significant differences in awareness by parental language, parental education, child gender, residential physical environment or number of adults in the family.

Parental awareness of the guidelines was not associated with parental co-participation in child DMU.

## 4. Discussion

In this study, we examined the DMU and physical play of children attending early childhood education centres in Finland, focusing specifically on parental co-participation and its sociodemographic correlates. Our data concerned children’s time outside of early childhood education, when the parental influence on children’s time use and developing health behaviours is greatest. The results showed that DMU takes up a significant part of children’s daily free time already at the age of two, and the time increases with age. Children used slightly less time for physically active outdoor play, and the time use was quite stable among all age groups. There appeared to be consistency in parental engagement in the activities, as co-participation in DMU and in physically active play correlated positively. On average, parents co-participated more in the child’s DMU than in their physical play. The younger the child, the more the parent co-participated for both activities. Parental co-participation varied with some sociodemographic factors. The highest co-participation rates for DMU were among parents who were under 30 years old, Finnish-speaking parents, those with no other children and those with a male child. For physical play, the highest rates were among fathers, parents under 30 years, Swedish-speaking parents, those with no other children, and those with a male child. In addition, we investigated the sociodemographic variation in parental awareness of the guidelines regarding co-participation in DMU. The groups most aware of the guidelines were mothers and parents belonging to the lowest income class. No association was found between awareness and co-participation.

Children spent 1.6 h on a weekday and 2.2 h on a weekend day on average using digital media, with no significant difference between genders. It is likely that the average total daily amount on weekdays is a little higher for the six-year-old children because of the use of digital media in pre-primary education [63]. The average times appear quite similar to the findings in other recent comparable studies conducted in Finland [19], Australia [64] and China [65], but they are significantly lower than in the US [20,66,67], as well as Greece [68], and a little lower compared to a recent Australian study [69]. The time use in the present study, as in most others, clearly exceeds the WHO guideline for daily sedentary time of one hour [70], a finding similar to that of a comparable study in Singapore [71]. However, all DMU may not be sedentary by nature [72]. Similar to other studies (e.g., [18,20,64]), we found that older children spent significantly more time using digital media than younger children. 

The average time spent in outdoor physical play was 1.3 h on a weekday and 1.8 h on a weekend day. The weekday times are not to be taken as the total daily amount of PA, as the measures concerned the time outside of early childhood education. A major proportion of children’s outdoor physical play on weekdays takes place in early childhood education in Finland [73], as also elsewhere [74], and 90% of the children in this dataset spent more than five hours a day in day care outside of the home. Moreover, the data were gathered during November, and cold weather has been associated with decreased PA in Finland [75], as well as in all of Northern Europe [76]. Comparisons between studies on PA require caution due to methodological differences [77] as, for example, parental observations may vary. Some recent studies in different parts of the world have yielded similar outdoor play times [65,78], but other studies have demonstrated much higher amounts [69,79]. Boys spent slightly more time on outdoor physical play than girls, which is a quite common phenomenon in Finland [80] and elsewhere [65,69,81]. There was no significant difference in time use based on child age. In Finland, one likely reason for this implied stability throughout early childhood is that daily outdoor play is a cultural norm [80]. The finding may also suggest that increased screen time may not directly lead to decreased physical play (see also [82]).

We found that parents co-participated more in children’s DMU than in their physically active play. There is lack of comparable research as, to the best of our knowledge, our study is the first to report on daily parental co-participation rates in these two activities using self-reported data and not separating PA by intensity. Other studies on approximately the same age group, using objective measures, have also found that parent–child joint daily activities are mostly sedentary by nature [44]. Earlier research on joint PA has shown that daily times spent in at least moderate-to-vigorous PA can be very low, only a couple of minutes [44,45]. In our study, the parent-reported co-participation rate in children’s physical play on a weekday was almost 30%, meaning much longer daily times than previously found. Possible interpretations are that the joint PA in our study was very low in intensity and that parents’ views on whether the play was physical and/or whether they were co-participating differ from objective measurements. Previous research has shown that co-participation for both PA [27,53,83] and DMU [41,53] is more common with a decreasing age of the child, and this was also evident in our results. Parental co-participation in children’s physical play decreased notably by age: on weekdays, parents co-participated in two-year-old children’s play twice as much as in six-year-old children’s play. The decrease was also substantial for co-participation in DMU, and this decrease was accentuated by the fact that the child’s time use for digital media seemed to increase with age. Parental co-participation was more than twenty percentage points higher for two-year-old children than for six-year-olds, meaning that an average parent co-participates only a third of the time as the child approaches school age. Co-participation in DMU and in physically active play correlated significantly, suggesting that co-participation may be a consistent parental practice, promoting health-enhancing behaviours in children. In addition, on weekends, there was significantly more parental co-participation in both activities. This suggests that parents’ co-participation is often hindered by daily schedules, an explanation offered by previous studies [41]. It is then worth noting that the majority of respondents were from two-parent families, and it is likely that both parents co-participate in the child’s activities [45,84], meaning that the rates per child are likely to be higher. The results on the daily parental time spent engaging in children’s activities are significant, as, through their behaviour, parents convey their attitudes, transmit healthy habits and, thus, have important impacts on their children’s health [19,21,23,24,25,30,31,32,35,36,37,38,39,40]. Consistency in co-participation in both activities may strengthen the positive health effects of co-participation for the child. 

Parental co-participation varied based on parental and child gender, parental age, parental language and family size. There were no significant differences in parental co-participation between groups with different educational or income levels or residential environment. The lack of socioeconomic differences seems somewhat surprising in light of recent studies [51,53,85]. However, caution is needed when drawing conclusions, as the participants in the current study had a higher average educational level than the population average. Nevertheless, differences between countries can also be explained by differences in, for instance, parental attitudes towards children’s DMU [53] and national policy measures [86]. Finnish parents across socioeconomic groups may share an exceptionally high level of trust in authorities and societal stakeholders in general [87] (p. 7) [88], and this may partially explain parents’ low levels of concern about their children’s screen time, for example, about watching a popular daily children’s television programme produced by the national public service media company, without parental mediation. A qualitative comparison with European countries has suggested that Finnish parents are the least worried about the risks of children’s DMU [51] (p. 17). However, cultural differences were found in co-participation, as parental language was a significant factor. First, co-participation in physical play was most evident among Swedish-speaking respondents. The Swedish-speaking population in Finland comprises a minority with tighter community relations and better health and life satisfaction than Finnish-speaking Finns [89]. These cultural characteristics may be associated with the differences in co-participation, and higher co-participation in physical play may even be one of the many factors producing the health differences. In terms of co-participation in DMU, there was a difference between the Russian- and Finnish-speaking parents; the Russian-speaking parents were the least active in co-participation. Previous qualitative research has also found that Finnish parents stand out among European parents as active mediators [85]. Among European parents, Russian parents were found to favour restrictive parenting practices and did not typically actively engage in children’s DMU [51]. These differences are likely to be related to more general norms and practices of parenting.

There were several gender-related differences in parental co-participation. Co-participation in boys’ DMU was more common than co-participation with girls. Research evidence has been somewhat contradictory; this finding is supported by an earlier study [41], but co-participation in sedentary behaviour has also been found to be more common with girls [53]. Previous research has stated that parents may be more concerned about the health effects of young boys’ DMU than of girls’ DMU [90], and this may partly explain the higher rate of co-participation in boys’ activities. It appears that parents of both genders equally participate in their children’s digital activities. However, co-participation in physical play was more common for fathers. This finding is consistent with previous research [47,49]. Finnish preschool-age children highly value the time spent together with their parents, especially with their mothers [91]; this suggests the importance of both parents’ co-participation in both activities.

Parental age explained co-participation, suggesting a generational difference. The youngest parents, born in the 1990′s, participated more than older parents. Previous studies [41,42] have implied that parental co-participation in children’s media use may be more usual in activities which are traditional or otherwise familiar to the parents. Younger parents have more knowledge on DMU, as well as its potential benefits and unfavourable consequences, and this may lead to greater efforts to mitigate DMU [51]. There is an intergenerational gap between parents’ and young children’s DMU due to digitalisation during recent decades, and active co-participation in children’s DMU may be challenging for parents in general [21,42]. This may be particularly significant in older parents. The age-related difference was revealed also in parental co-participation in physical play. A smaller number of children of any age was a significant determinant of co-participation in both activities. Fewer children may mean more time for one child. Nevertheless, previous studies have found that siblings’ influence on the needs and possibilities of parental co-participation can vary depending on the children’s age range [46,92,93]. For example, older children can help parents to mitigate younger children’s DMU [85] (p. 17). Interestingly, the number of adults in the family was not a significant factor. In other words, single parents co-participated in children’s DMU and physical play as much as parents in two-parent families. Previously, it has been reported that children in single-parent families use digital media more on their own than children in two-parent families [50]. These contradictions imply that more research is needed on how and why the family structure influences parental co-participation (see also [37]). Conclusions on the potentially significant role of grandparents could not be drawn in this study, due to the small number of respondents who were grandparents.

Fewer sociodemographic differences were found in the awareness of the guidelines recommending co-participation than in daily co-participation. Those in the middle income level (EUR 40,000–69,000) were less aware of the guideline than those in the lowest income group. A higher awareness in the lowest income group is somewhat surprising, but nearly similar findings have been reported regarding parental attitudes toward screen time [94]. In addition, awareness did not directly decrease with income, so caution is needed when interpreting the data. Similar to previous findings [95], women were more aware than men. Interestingly, there was no difference between genders in the daily co-participation rate. In addition, awareness was not associated with co-participation. This finding is surprising as it contradicts previous research regarding screen time and sedentary time guidelines [96,97].

When the findings are applied to the FEM theoretical framework [48], they stress the importance of looking at family structure and other family ecological factors that form the context for parental co-participation. As research has shown favourable health effects of co-participation in DMU [19,21,36,37,38,39,40] and PA [30,31,32,35], the ecological factors recognised in this study as correlates of co-participation may be significant for children’s health and development and contribute to sociodemographic differences in health. Parental awareness of the guidelines was not associated with following these guidelines in daily life, which implies that multiple contextual factors may hinder the ability to act according to one’s knowledge and beliefs. Nevertheless, the sociodemographic factors which correlated with co-participation are likely to have a positive influence on the social and emotional components in the FEM and, thus, enable co-participation. For health promotion in practice, evidence-based guidance, such as guidelines on parental co-participation and on a daily balance between PA and DMU, is needed [21], as well as in parents’ views [98]. However, the findings reveal the need to take into account the sociodemographic differences that influence parents’ abilities and needs to co-participate. Increases in parental co-participation are most likely to be achieved when contextual factors, including the sociodemographic factors investigated in this study, favour behavioural change. This study examined individual associations, and their impact may vary depending on the presence of other contextual factors [46]. A broad look at unique life circumstances is needed in efforts to improve parents’ abilities to promote healthy habits in their young children (see also [95,99]). With increasing digitalisation, parents’ abilities to actively co-participate in children’s DMU and PA are both increasingly challenged and increasingly important [21,42].

Our study had several strengths. The sample was nationally representative and of adequate size. The survey used the online platform, Qualtrics, which has been reported to be suitable and effective for collecting self-reported or proxy data in health-related research [100]. Participation was voluntary. To reduce social desirability and recall bias, respondents could fill the questionnaire anonymously on the secure online platform, the recall was limited to no more than seven days, and the questionnaire instructions stated that there were no right or wrong answers. In addition, the validity of the questionnaire has been tested beforehand [58]. The current study also had some limitations. In this study, causal relationships could not be identified. Further interventional studies and longitudinal follow-ups would be required to investigate whether certain sociodemographic factors determine certain parental behaviours and how strong the causal relationships are. Importantly, such studies could also reveal possible two-way causalities, in which behavioural factors strengthen the existing social and structural differences between families. Scientific knowledge on these kinds of relationships can be useful in designing programmes aimed at behavioural change. Information on who did not participate in the study did not exist, as, after controlling the participant municipalities and day care centres, there was no ability to control who eventually participated the study. The participation rate was low, the participants had relatively high socioeconomic statuses, and the interruption rate for response was relatively high. A small proportion of parents did not use the electronic communication system provided by the early childhood education centres and, thus, may have been unable to participate regardless of the offered paper information letter and paper survey alternative.

In this study, parental participation in children’s DMU and physically active play were measured via only one parent per child, and 89% of respondents had more than one adult in their family. The whole family influences the development of children’s health behaviours [22], and the number of adults and children in the family undoubtedly has an effect on the duration of time that the respondent parent is able to engage in their child’s DMU and physical play. We accounted for family structure in the analyses by including the variables, number of children and number of adults living in the household, as explaining factors of parental co-participation in the models. However, other guardians’ participation in children’s activities remained unknown in this study, and further studies are needed to examine the total co-participation per child. An earlier study in Finland has shown that parents’ self-reported parenting practices regarding physical activity, including co-participation, are strongly associated with their partners’ practices [84]. This implies that results gathered from one parent may shed light on co-participation and its correlates with regard to the other parent in the family. It is also important to question the extent to which the results are applicable outside of the Finnish cultural context. For instance, there are differences in family structures among different cultures and cultural differences in the role of, for example, grandparents, in children’s daily lives. Considering the low number of respondents who were grandparents (one grandmother and two grandfathers), it is most likely that our dataset fairly represents the relatively narrow Finnish concept of a family, most often considered to consist of only parents and children, in other words, a nuclear family. Further studies could also investigate the role of the factors in the FEM which were not included in this study. Up-to-date research is also needed on the impact of the COVID-19 pandemic on DMU and PA, as well as on the association between parental co-participation and children’s objectively measured PA. Research in other countries may also reveal culture- and country-specific factors and further explain parental co-participation.

## 5. Conclusions

According to our results, the daily lives of young children become increasingly digitalised. In their DMU and physically active play at home, they are only partly accompanied by an actively engaging parent. Parental co-participation varied based on parental and child gender, parental age, parental language and family size. There were no significant differences in parental co-participation based on educational or income level or residential environment. As parental co-participation has an impact on the health consequences of DMU, as well as on the development of health behaviours in children, the results concerning sociodemographic differences in co-participation are significant for health promotion. Health care personnel should encourage parents to co-participate with their children, and recommendations for parents regarding co-participation are needed. Interventions should address multiple sociodemographic factors that influence parental co-participation and adherence to guidelines. The sociodemographic groups identified in this study may need special support and guidance in order to promote equality in families’ abilities to foster healthy lifestyles.

## Figures and Tables

**Table 1:** Descriptive characteristics of the participants (*n* = 2512).

Variable	Sample N (%)	Valid N (%)	Mean Age (SD)
Parent gender			
Woman	1510 (60.1)	1510 (83.7)	35.8 (5.23)
Man	284 (11.3)	284 (15.7)	38.4 (5.88)
Missing	718 (28.6)	-	-
Total	2512 (100)	1794 (100)	36.2 (5.42)
Child gender			
Girl	874 (34.8)	874 (48.3)	4.75 (1.38)
Boy	933 (37.1)	933 (51.6)	4.67 (1.37)
Other	2 (0.1)	2 (0.1)	4.00 (0.28)
Missing	703 (28.0)	-	-
Total	2512 (100)	1809 (100)	4.71 (1.37)
Language			Population ^a^ %
Finnish	2257 (89.2)	2257 (89.2)	87.3
Swedish	167 (6.6)	167 (6.6)	5.2
Russian	54 (2.1)	54 (2.1)	1.5
English	51 (2.0)	51 (2.0)	6.0
Parental education			
Elementary/No formal education	46 (1.8)	46 (2.6)	14
Secondary	91 (3.6)	91 (5.1)	49 ^b^
Vocational	437 (17.4)	437 (24.3)	-
Bachelor’s degree	669 (26.6)	669 (37.2)	21
Master’s degree	553 (22.0)	553 (30.8)	16
Missing	716 (28.5)	-	-
Total	2512 (100)	1796 (100)	-
Household income (EUR)			
0–13,999	78 (3.1)	78 (4.4)	
14,000–19,999	65 (2.6)	65 (3.7)	
20,000–39,999	273 (10.9)	273 (15.4)	
40 000–69,999	598 (23.8)	598 (33.8)	
70,000–99,999	490 (19.5)	490 (27.7)	
100,000–119,999	129 (5.1)	129 (7.3)	
120,000 or more	138 (5.5)	138 (7.8)	
Missing	741 (29.5)	-	
Total	2512 (100)	1771 (100)	
Living environment			
Urban	978 (38.9)	978 (57.3)	72
Rural	729 (29.0)	729 (42.7)	28
Missing	805 (32.0)	-	-
Total	2512 (100)	1716 (100)	-
No. of adults in family			
One	194 (7.7)	194 (11)	22
Two or more	1594 (63.5)	1594 (89)	78
Missing	724 (28.8)	-	-
Total	2512 (100)	1788 (100)	-
No. of children aged 0–6			
One	1093 (43.5)	1093 (61)	
Two	629 (25.0)	629 (35)	
Three or more	67 (2.7)	67 (3)	
Missing	723 (28.8)	-	
Total	2512 (100)	1789 (100)	
No. of children aged 7–18			
Zero	1140 (45.4)	1140 (63)	
One	472 (18.8)	472 (26)	
Two or more	191 (7.6)	191 (10)	
Missing	709 (28.2)	-	
Total	2512 (100)	1803 (100)	

^a^ Population of Finland in 2019 [62]. Education level from the population of 25–49 year-olds living in Finland. ^b^ Secondary and vocational together as secondary level degree.

**Table 2:** Children’s digital media use (DMU) and outdoor physical play by child’s age.

Variable	Child’s Age	Weekday Mean (SD)	*p*	Weekend Mean (SD)	*p*
Child DMU h/day			0.019		<0.001
	2 years	1.41 (1.34)		1.79 (1.20)	
	3 years	1.51 (1.34)		2.02 (1.35)	
	4 years	1.54 (1.30)		2.17 (1.21)	
	5 years	1.57 (1.20)		2.34 (1.49)	
	6 years	1.74 (1.43)		2.57 (1.61)	
Child Outdoor Physical Play h/day			0.906		0.723
	2 years	1.24 (1.14)		1.69 (0.88)	
	3 years	1.27 (1.16)		1.72 (0.99)	
	4 years	1.27 (1.27)		1.79 (1.04)	
	5 years	1.26 (1.49)		1.77 (1.07)	
	6 years	1.18 (1.56)		1.78 (1.22)	

**Table 3:** Parental co-participation in child’s digital media use (DMU) and physical play by child’s age.

Variable	Child’s Age	Weekday Mean (SD)	*p*	Weekend Mean (SD)	*p*
Co-Participation in DMU %			<0.001		<0.001
	2 years	57.2 (30.6)		60.3 (29.4)	
	3 years	45.3 (29.1)		50.7 (29.0)	
	4 years	40.9 (29.6)		46.8 (28.9)	
	5 years	36.4 (27.5)		41.7 (26.8)	
	6 years	30.5 (25.4)		38.0 (25.4)	
Co-Participation in Physical Play %			<0.001		<0.001
	2 years	43.1 (30.6)		56.6 (28.7)	
	3 years	34.3 (27.2)		48.1 (28.1)	
	4 years	28.1 (24.9)		42.4 (26.8)	
	5 years	25.8 (24.8)		39.9 (27.5)	
	6 years	20.2 (21.9)		32.9 (25.0)	

**Table 4:** Odds ratios (ORs) of sociodemographic variables to parental co-participation in child’s digital media use with 95% confidence interval (CI).

Variable	OR	95% CI	*p*
Language			0.010
Finnish	1	reference	
English	0.762	(0.371, 1.545)	
Russian	0.314	(0.149, 0.632)	
Swedish	0.872	(0.587, 1.292)	
Parent gender			0.916
Male	1	reference	
Female	0.986	(0.756, 1.286)	
Parent age	0.973	(0.955, 0.992)	0.005
Household income (EUR)			0.529
0–13,999	1	reference	
14,000–19,999	1.168	(0.601, 2.266)	
20,000–39,999	0.931	(0.555, 1.567)	
40,000–69,999	0.980	(0.590, 1.631)	
70,000–99,999	1.033	(0.607, 1.761)	
100,000–119,999	0.724	(0.390, 1.345)	
120,000 or more	0.776	(0.414, 1.455)	
Parental education			0.468
Elementary/No formal education	1	reference	
Secondary	0.804	(0.394, 1.642)	
Vocational	0.720	(0.389, 1.335)	
Bachelor’s degree	0.701	(0.380, 1.295)	
Master’s degree	0.613	(0.327, 1.150)	
Child gender			0.038
Boy	1	reference	
Girl	0.821	(0.682, 0.989)	
Home environment			0.763
Urban	1	reference	
Rural	1.030	(0.850, 1.248)	
No. of adults			0.534
One	1	reference	
Two or more	1.114	(0.792, 1.570)	
No. of children aged 0–6			<0.001
One	1	reference	
Two	0.659	(0.535, 0.811)	
Three or more	0.449	(0.272, 0.734)	
No. of children aged 7–18			<0.001
Zero	1	reference	
One	0.415	(0.329, 0.522)	
Two or more	0.366	(0.262, 0.509)	

**Table 5:** Odds ratios (OR) of sociodemographic variables to parental participation in child’s physical play.

Variable	OR	95% CI	*p*
Language			0.029
Finnish	1	reference	
English	1.727	(0.868, 3.423)	
Russian	0.938	(0.470, 1.826)	
Swedish	1.685	(1.137, 2.491)	
Parent gender			0.001
Male	1	reference	
Female	0.620	(0.474, 0.812)	
Parent age	0.943	(0.925, 0.962)	<0.001
Household income (EUR)			0.664
0–13,999	1	reference	
14,000–19,999	0.804	(0.405, 1.593)	
20,000–39,999	0.896	(0.530, 1.517)	
40,000–69,999	0.829	(0.496, 1.391)	
70,000–99,999	0.924	(0.541, 1.588)	
100,000–119,999	0.930	(0.499, 1.739)	
120,000 or more	1.234	(0.653, 2.338)	
Parental education			0.236
Elementary/No formal education	1	reference	
Secondary	0.712	(0.346, 1.464)	
Vocational	0.678	(0.366, 1.261)	
Bachelor’s degree	0.714	(0.387, 1.323)	
Master’s degree	0.560	(0.298, 1.056)	
Child gender			0.020
Boy	1	reference	
Girl	0.797	(0.657, 0.965)	
Home environment			0.240
Urban	1	reference	
Rural	1.127	(0.923, 1.375)	
No. of adults			0.440
One	1	reference	
Two or more	0.872	(0.616, 1.235)	
No. of children aged 0–6			<0.001
One	1	reference	
Two	0.555	(0.447, 0.689)	
Three or more	0.322	(0.186, 0.544)	
No. of children aged 7–18			<0.001
Zero	1	reference	
One	0.546	(0.430, 0.692)	
Two or more	0.443	(0.312, 0.624)	

**Table 6:** Odds ratios (ORs) of sociodemographic variables to parental awareness of the guidelines regarding co-participation in child’s digital media use.

Variable	OR	95% CI	*p*
Language			0.097
Finnish	1	reference	
English	0.816	(0.372, 1.87)	
Russian	1.876	(0.796, 5.18)	
Swedish	1.685	(1.019, 2.907)	
Parent gender			<0.001
Male	1	reference	
Female	2.392	(1.78, 3.215)	
Parent age	1.011	(0.988, 1.034)	0.343
Household income (EUR)			0.006
0–13,999	1	reference	
14,000–19,999	0.449	(0.176, 1.104)	
20,000–39,999	0.716	(0.321, 1.47)	
40,000–69,999	0.424	(0.194, 0.85)	
70,000–99,999	0.491	(0.219, 1.01)	
100,000–119,999	0.848	(0.346, 1.966)	
120,000 or more	0.563	(0.231, 1.287)	
Parental education			0.326
Elementary/No formal education	1	reference	
Secondary	0.714	(0.276, 1.74)	
Vocational	0.728	(0.311, 1.557)	
Bachelor’s degree	0.586	(0.252, 1.245)	
Master’s degree	0.738	(0.312, 1.601)	
Child gender			0.068
Boy	1	reference	
Girl	0.813	(0.65, 1.016)	
Home environment			0.257
Urban	1	reference	
Rural	0.876	(0.697, 1.101)	
No. of adults			0.738
One	1	reference	
Two or more	0.93	(0.601, 1.42)	
No. of children aged 0–6			0.079
One	1	reference	
Two	1.01	(0.789, 1.295)	
Three or more	0.532	(0.305, 0.935)	
No. of children aged 7–18			0.169
Zero	1	reference	
One	1.189	(0.907, 1.565)	
Two or more	1.411	(0.948, 2.134)	
